# Pore-Fiber Transport Dynamics of Aqueous Cosolvent Solutions in Paper

**DOI:** 10.1021/acs.langmuir.4c01965

**Published:** 2024-09-03

**Authors:** Sajjad Karimnejad, Elian Gonnet, Shuo Wang, Hamid Mansouri, Nicolae Tomozeiu, Anton A. Darhuber

**Affiliations:** † Fluids & Flows Group, Department of Applied Physics, Eindhoven University of Technology, 5600MB Eindhoven, The Netherlands; ‡ Canon Production Printing, 5914HH Venlo, The Netherlands

## Abstract

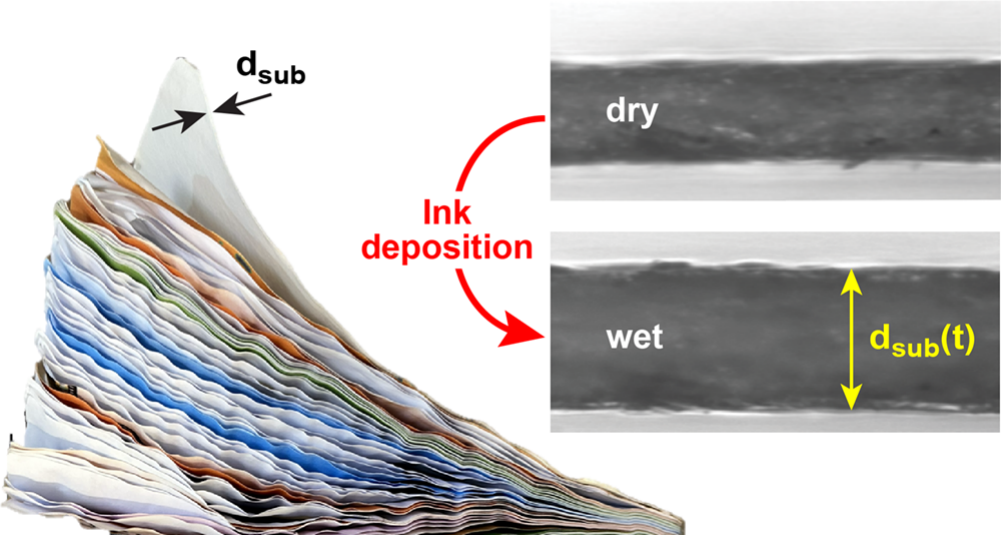

After inkjet printing onto uncoated and unsized paper, the ink is first imbibed into the interfiber pores and subsequently absorbed by the cellulose fibers. The achievable print quality depends on the rate of this pore-fiber transport. The latter is accompanied by mechanical expansion of the fibers and the paper sheet. Therefore, we systematically monitored the swelling dynamics of several paper types as a function of ink composition by means of four different measurement techniques. Using aqueous cosolvent solutions as model inks, we found an approximately exponential relation of the time scales of pore-fiber transport with the cosolvent concentration and an approximately linear relation with its molecular weight. Addition of surfactants can substantially speed-up pore-fiber transport.

## Introduction

Inkjet printing technology has numerous applications ranging from flexible electronics^1^ to the graphical printing industry.^2^ For printing on paper, water-based inks are an environmentally friendly option. In terms of weight fraction, the two main constituents of aqueous inkjet printing inks are typically water as the primary solvent and polar liquids of low volatility such as glycerol or poly(ethylene glycols). The latter are called cosolvents and make up 5–50% of the ink volume.^3^ They serve several purposes, most importantly as humectants to prevent inkjet nozzle clogging.^4^

Pore-fiber transport^5−8^ of the liquid-phase ink components is an important factor in inkjet printing. It influences how close to the surface of a paper sheet the colorant pigments will be immobilized, thereby affecting color depth. Moreover, it influences the lateral spreading of an ink dot and thus the achievable resolution. An important aspect is how the rate of fiber absorption compares to the drying time in an inkjet printer, which typically ranges from 1 to 10 s.

Paper consists mainly of fibers, which are composed of cellulose fibrils.^9^ These fibrils contain amorphous and crystalline domains in a proportion that critically depends on the chemical and mechanical processes applied during paper making.^10,11^ There is consensus that it is primarily the amorphous domains that can accommodate solvent molecules and swell in the process.^12−19^

Water-induced swelling of thin cellulose-based porous media and single fibers has been studied extensively.^13,20−23^ Letková et al.^24^ measured a swelling time of roughly 1 s for never-recycled paper sheets made of bleached hardwood kraft pulp with a short-time swelling amplitude (i.e., a relative increase in thickness) of about 60%.^24^ After seven recycling steps, the swelling time increased to 1.75 s and the short-time swelling amplitude decreased to 40%. They observed a fast (order 1 s) as well as a slow swelling process (order 1 h). Schuchardt and Berg performed swelling measurements of a single carboxymethyl cellulose fiber and reported a short swelling time of 15–60 s and a long one of the order of 10 min.^25^ Geffert et al.^26^ and Jablonskỳ et al.^27^ found analogous results using hand sheets of bleached sulfate pulp composed of a blend of hardwood species and commercial newsprint paper, respectively.

In this manuscript, we determined pore-fiber transport rates of aqeuous cosolvent solutions in commercial paper substrates by monitoring the transient swelling of the paper sheets after droplet deposition. Due to the low stiffness of wet paper, we employed four optical noncontact techniques: white light interferometry (WLI), microscopy-based thickness monitoring, laser triangulation, and confocal microscopy. We measured the expansion strain in the thickness direction (TD) as a function of time and systematically varied the cosolvent composition and concentration. Moreover, we studied the influence of various anionic and nonionic surfactants.

## Experimental Section

### Materials and Material Properties

As substrates, we used the following uncoated, uncalendered and unsized papers from three different manufacturers:

- paper ‘A’ (grammage *g* = 80g/m^2^, thickness *d*_sub_ = 104 μm).
- paper ‘B’ (*g* = 90g/m^2^, *d*_sub_ = 116 μm, ash content 12%).
- paper ‘C’ (*g* = 80g/m^2^, *d*_sub_ = 180 μm, made from cotton linters, ashless).

Papers A and B are commercial printing papers, whereas paper C is a commercial filter paper. Judging by the much larger value of *d*_sub_ for the same value of *g*, paper C is subject to less compression during manufacture than paper A. Our selection of uncoated, uncalendered and unsized paper types is further motivated in the Discussion section. To protect the papers from changes in ambient conditions, they are stored in a sealed container until each experiment.

The cosolvents listed in Table 1 were purchased from Sigma-Aldrich and used as received. We selected them because they are nontoxic, environmentally friendly and industrially relevant. Moreover, PEG offers the key benefit that oligomers of different molecular weight (MW) are commercially available in high purity grades. This allows to tune the ratio of the molecular size and the intrafiber poresize of the cellulose fibers. Aqueous cosolvent solutions were prepared by mixing deionized water (Millipore Direct-Q3 R) and a pure cosolvent at a certain mass ratio in a glass bottle. Masses were measured with a precision scale (Kern, ALT 220-5DAM). For some experiments, we added the anionic surfactants sodium dodecyl sulfate (SDS) and sodium lauroyl sarcosinate (SLS) to the cosolvent solutions or the nonionic surfactants Triton X-100 (TX-100) and Brij30, see Table 1. Their chemical structures are presented as insets in Figure 6. For one experiment, we used hexadecane (Sigma-Aldrich product number H6073, MW = 226 g/mol).

**Table 1:** List of Cosolvents and Surfactants Including Sigma-Aldrich Product Numbers and Molecular Weights (MWs)

cosolvent/surfactant	product#	MW
glycerol	449770	92.1
ethylene glycol (EG)	324558	62.1
diethylene glycol (DEG)	32160	106.1
triethylene glycol (TrEG)	90390	150.2
tetrathylene glycol (TEG)	110175	194.2
polyethylene glycol 300 (PEG6)	49770	300 ± 15
Brij30	235989	362
Triton X-100 (TX-100)	T9284	647
sodium dodecyl sulfate (SDS)	436143	288.4
sodium lauroyl sarcosinate (SLS)	61744	293.4

We used a concentric-cylinder viscometer (Brookfield II+Pro) to measure viscosities and a Wilhelmy plate (NIMA, PTP-10) together with a precision scale (Kern, ALT 220-5DAM) for measuring surface tensions of cosolvent solutions.

### Experimental Setups and Procedures

In this section, the methods utilized for monitoring pore-fiber transport will be discussed in detail. All experiments were conducted at room temperature (298 ± 2) K and an ambient relative humidity of (40 ± 10)%. Paper ‘A’ is typically used for all the experiments, unless otherwise stated. Information pertaining to laser triangulation and confocal microscopy can be found in the Supporting Information (Section II and Figures S2–S4).

#### White-Light Interferometry (WLI)

We used a Bruker NPFLEX white light interferometer with a 5× objective lens and a 0.55× demagnification lens, resulting in a field of view of approximately 2 mm. The built-in camera has 640 × 480 pixels. The paper samples are placed horizontally, i.e., parallel to the *xy*-plane. The objective lens is scanned along the vertical *z*-direction over a range of approximately 90 μm with a scan rate of approximately 25 μm/s. This results in a time resolution of approximately 3.6 s. For every camera pixel, corresponding to a specific (*x*,*y*)-position on the sample, the *z*-position corresponding to zero optical path difference relative to a reference beam is obtained.^28,29^

To prevent the paper from curling and bulging and to keep it flat and in contact with its support surface during an experiment, samples were placed on a suction plate. The plate comprises a hole array (hole diameter 100 μm, array period 200 μm, array dimensions 4 × 4 mm^2^) that is connected to a vacuum cavity and an oil-free membrane pump. The underpressure was set to the minimum required to keep the paper flat using a needle valve between the pump and the cavity. In this fashion, the change in the average *z*-coordinates of the top surface of the sample Δ*z* reflects its thickness expansion.

#### Microscopy-Based Thickness Monitoring

**Figure 1:**
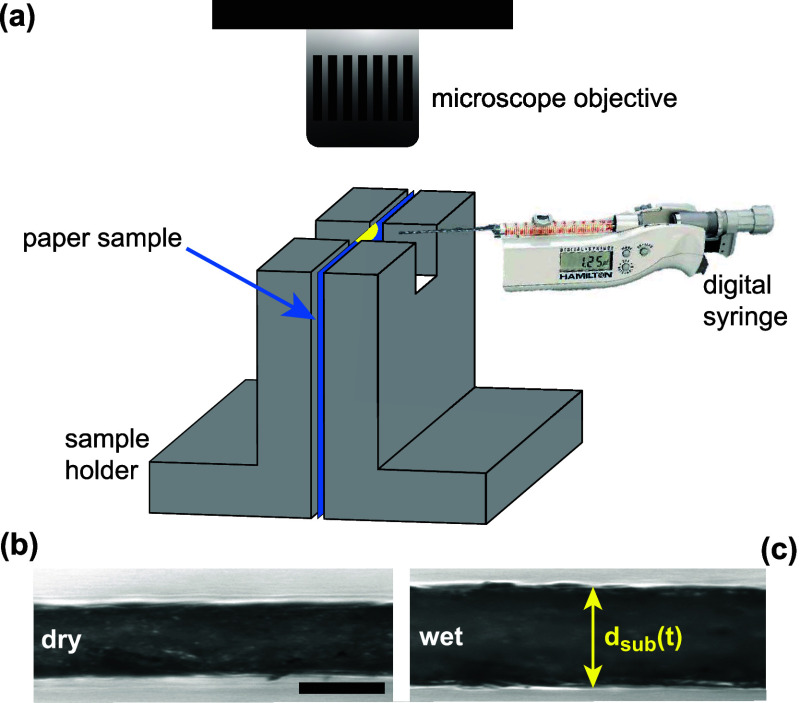
(a) Experimental setup for microscopy-based paper thickness monitoring. (b,c) Top-view of a paper sheet (b) before and (c) after liquid deposition. The scale bar in (b) corresponds to 100 μm.

We used the same method employed in the past e.g., by Lee et al.,^23^ Chang and Kim,^30^ and Kvick et al.^31^Figure 1a illustrates the setup for microscopy-based paper thickness monitoring. A sheet of paper is supported in a vertical orientation by two anodized metal holders under an upright microscope (Olympus BX50) with a 10× objective. The microscope is equipped with a CCD camera (Basler piA1000-60gm, 1004 × 1004 pixels, field of view 710 × 710 μm) and records a cross-section view of a paper sheet to monitor its thickness change. At the top of the metal holders, there is a recess (dimensions 5 × 5 mm^2^), through which model ink can be deposited onto the paper substrate. We used a Hamilton digital syringe to deposit droplets of volume *V* = 1 μL. The volume should be as small as possible to minimize the droplet imbibition time (≈ 1 s for *V* = 1 μL of pure water), but large enough to keep the reproducibility of *V* sufficiently high and the diameter of the wet zone (typically^6^ 4–5 mm) larger than the cellulose fiber length (typically^32^ 1–2 mm) (see Supporting Information, Figure S7). The metal holders keep the paper sample as flat as possible and minimize movements of the paper sheet during droplet deposition and swelling. The paper sheets are oriented with their machine direction parallel to the optical axis of the microscope to minimize buckling-induced displacements.^7^

## Results and Discussion

The thickness increase of the paper sheets after droplet deposition is quantified in terms of the expansion strain in the thickness direction
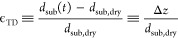
1where *d*_sub_(*t*) denotes the time-dependent thickness of the paper sheet after cosolvent deposition and *d*_sub,dry_ that of dry paper, i.e., prior to liquid deposition. The parameter Δ*z* ≡ *d*_sub_(*t*) – *d*_sub,dry_ corresponds to the thickness expansion.

### White-Light Interferometry

**Figure 2:**
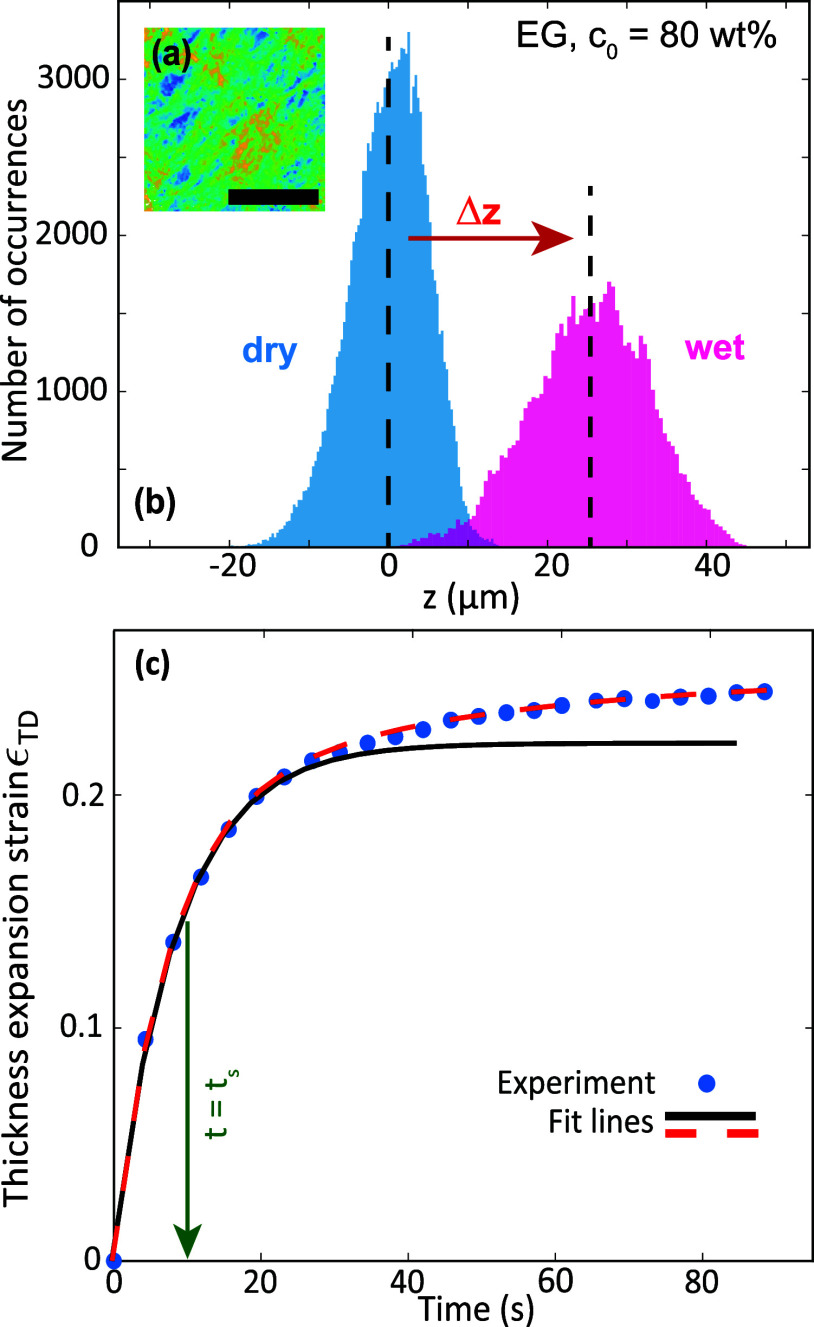
(a) Pseudocolor plot of the WLI raw data *z*(*x*,*y*) of dry paper A. The color range from dark blue to yellow represents a height variation of 34 μm. The scale bar corresponds to 0.75 mm. (b) Histograms of *z*-coordinates for paper A before (blue) and 87 s after (magenta) deposition of an EG solution droplet (*c*_0_ = 80 wt %). The vertical dashed lines indicate the mean values of the distributions. (c) Thickness expansion strain ϵ_TD_ as a function of time for an aqueous EG solution (*c*_0_ = 80 wt %, circles). The solid (black) and dashed (red) lines are fits according to eqs 2 and 3, respectively.

The raw WLI data consist of 640 × 480-sized matrices with *z*(*x*,*y*)-coordinates that represent the topography of the paper surface. An example is shown in Figure 2a. Figure 2b shows corresponding histograms of *z*-coordinates obtained with a sample of paper A before and after deposition of an EG solution droplet (*c*_0_ = 80 wt %). These histograms reflect the surface roughness of a paper sheet and represent a sample area of approximately 1.5 × 2 mm^2^. The bin sizes for the dry and wet sample were chosen to be 0.3 and 0.5 μm, respectively. The vertical dashed lines represent the mean values of the distributions. Their difference Δ*z* equals the average thickness expansion of the sample. Figure 2c depicts the time evolution of thickness expansion strain ϵ_TD_ for the same experiment. A rapid increase in Δ*z* is observed in the first 25 s, after which expansion continues at a much slower rate. The black solid line is a fit according to a single exponential fit function
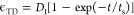
2which represents the first 25 s of the data very well. The numerical values of the fit parameters are *D*_1_ = 0.22 and *t*_s_ = 8 s. The parameter *t*_s_ quantifies the short swelling time, i.e., when the swelling amplitude reaches 63% of its asymptotic value.

Since the data for *t* > 30 s do not conform to a single exponential, we also performed a fit according to a double exponential fit function
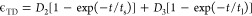
3which is represented by the red dashed line in Figure 2c. Here, *D*_2,3_, *t*_s_ and *t*_l_ are fit parameters with numerical values 0.191, 0.06, 7.4, and 37 s, respectively. The double exponential function results in an excellent fit over the entire data range, but yields essentially the same value of *t*_s_ as the single-exponential fit.

### Microscopy-Based Thickness Monitoring

**Figure 3:**
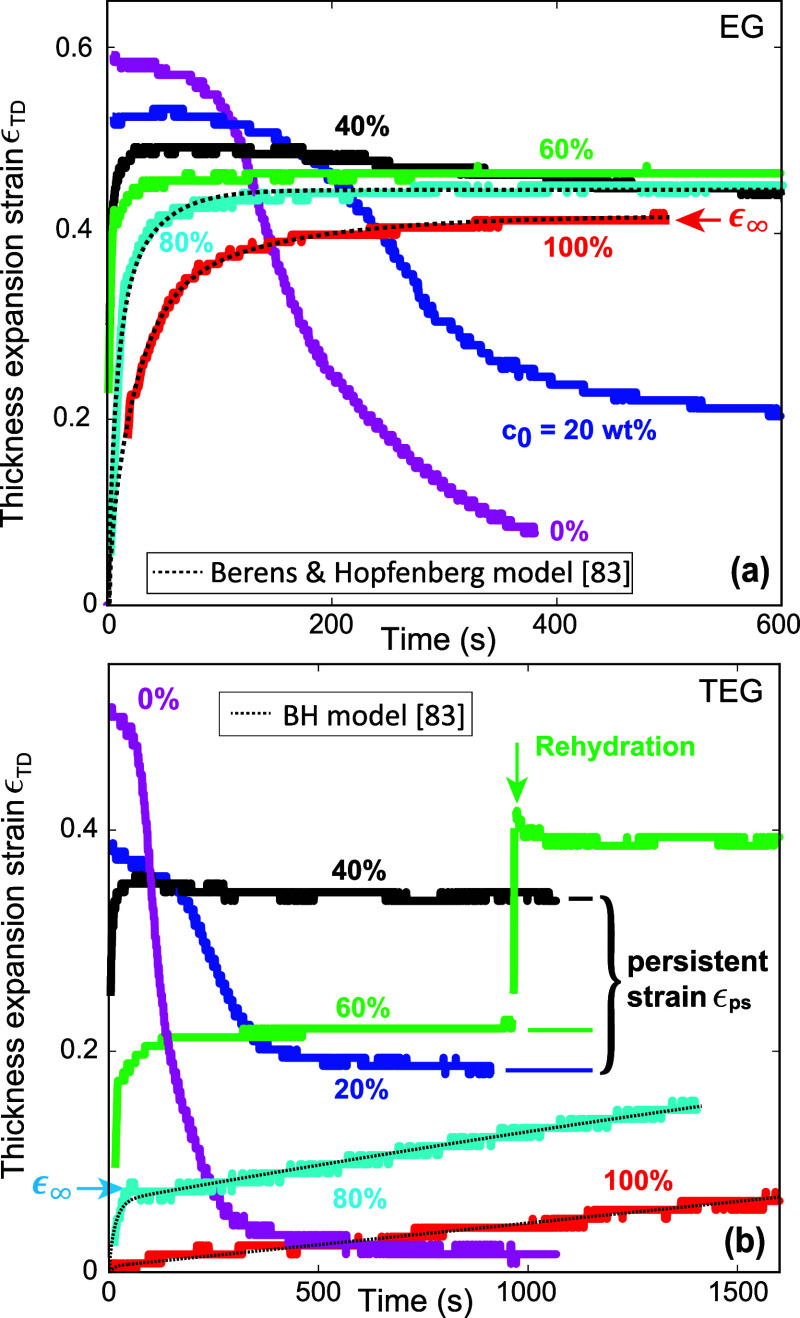
Thickness expansion strain ϵ_TD_ as a function of time for (a) EG and (b) TEG solutions and different values of *c*_0_. The dotted lines are fits according to the Berens and Hopfenberg (BH) model.

Figure 3a,b shows the thickness expansion strain ϵ_TD_ after droplet deposition as a function of time for EG and TEG solutions, respectively, and different values of *c*_0_. For pure water, the swelling occurs within the first few seconds, whereas it takes about 22 s for pure EG and considerably longer for pure TEG. For the data shown in Figure 3 and all subsequent figures, we define the short swelling time *t*_s_ as the instant when the expansion strain reaches 63% of an asymptotic value ϵ_*∞*_. As an example, the chosen value of ϵ_*∞*_ for pure EG is indicated by a red arrow. For most curves, ϵ_*∞*_ corresponds to the maximum value of ϵ_TD_. However, the curve corresponding to an 80 wt % TEG solution in Figure 3b clearly exhibits more than one time scale, which requires a different choice for ϵ_*∞*_, as indicated by the blue horizontal arrow.

A notable feature in Figure 3a,b is the presence of persistent strain ϵ_ps_ , i.e., the fact that the paper thickness does not return to its original dry value.^7^ This is due to the fact that cosolvents are essentially nonvolatile on the time scale of our experiments. Consequently, the fraction of the deposited cosolvents that are transported into the fiber interior, induces a certain remanent level of fiber swelling even after the water has evaporated. For both EG and TEG, the persistent strain ϵ_ps_ increases with *c*_0_ up to a maximum value *c*_0,crit_ beyond which it decreases. For EG and TEG the values of *c*_0,crit_ are approximately 60 and 40 wt %, respectively.

**Figure 4:**
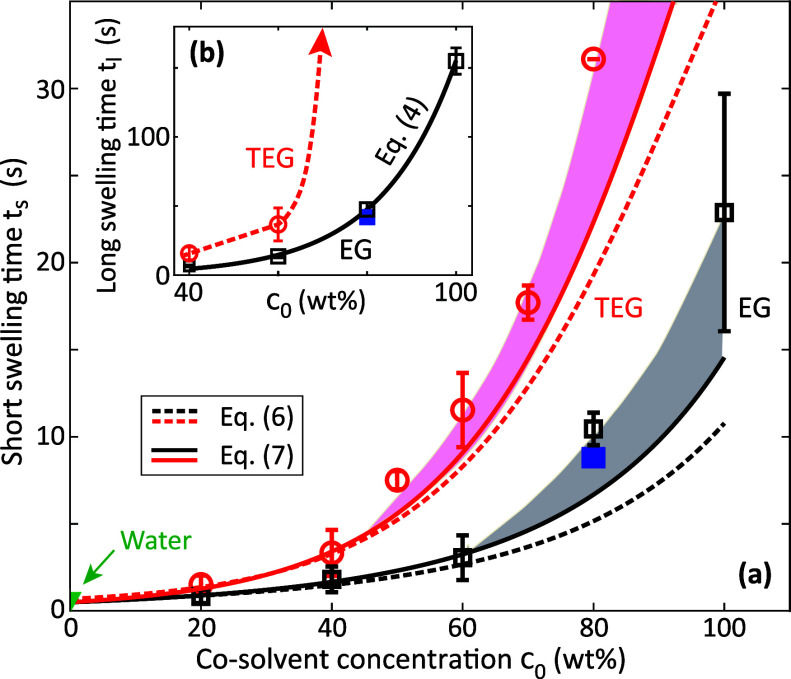
(a) Short swelling time *t*_s_ of paper A as a function of initial concentration *c*_0_ of EG (black squares) and TEG (red circles) solutions. The dashed and solid lines represent fitfunctions according to eqs 6 and 7, respectively. (b) Long swelling time *t*_l_ of paper A as a function of *c*_0_ for EG (black squares) and TEG (red circles) solutions. The black solid line represents an exponential fit-function according to eq 4. The dashed solid line is a guide to the eye. Open symbols in (a,b) correspond to microscopy-based thickness expansion data, the solid blue squares to the WLI experiment in Figure 2.

Figure 4a shows the short swelling time *t*_s_ as a function of the initial concentration *c*_0_ for EG and TEG solutions. Open symbols correspond to microscopy-based thickness expansion data and the filled square to the WLI experiment from Figure 2, demonstrating the excellent agreement between the two experimental methods. Analogous data for DEG are presented in Figure S8 of the Supporting Information.

Figure 4b shows the long swelling time *t*_l_ as a function of *c*_0_ for EG and TEG solutions. The black line in Figure 4b corresponds to the fit function
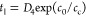
4with fit-parameters *D*_4_ = 0.42 s and *c*_c_ = 17 wt %, which represents the experimental data very well. For TEG, a drastic increase in *t*_l_ by more than 2 orders of magnitude is observed at a concentration *c*_0_ = (70 ± 10) wt %, see Table 2.

**Table 2:** Long Swelling Time *t*_l_ of Aqueous TEG Solutions as a Function of *c*_0_

*c*_0_ (wt %)	*t*_l_ (s)
60	37 ± 10
80	>6000
100	>10,000

**Figure 5:**
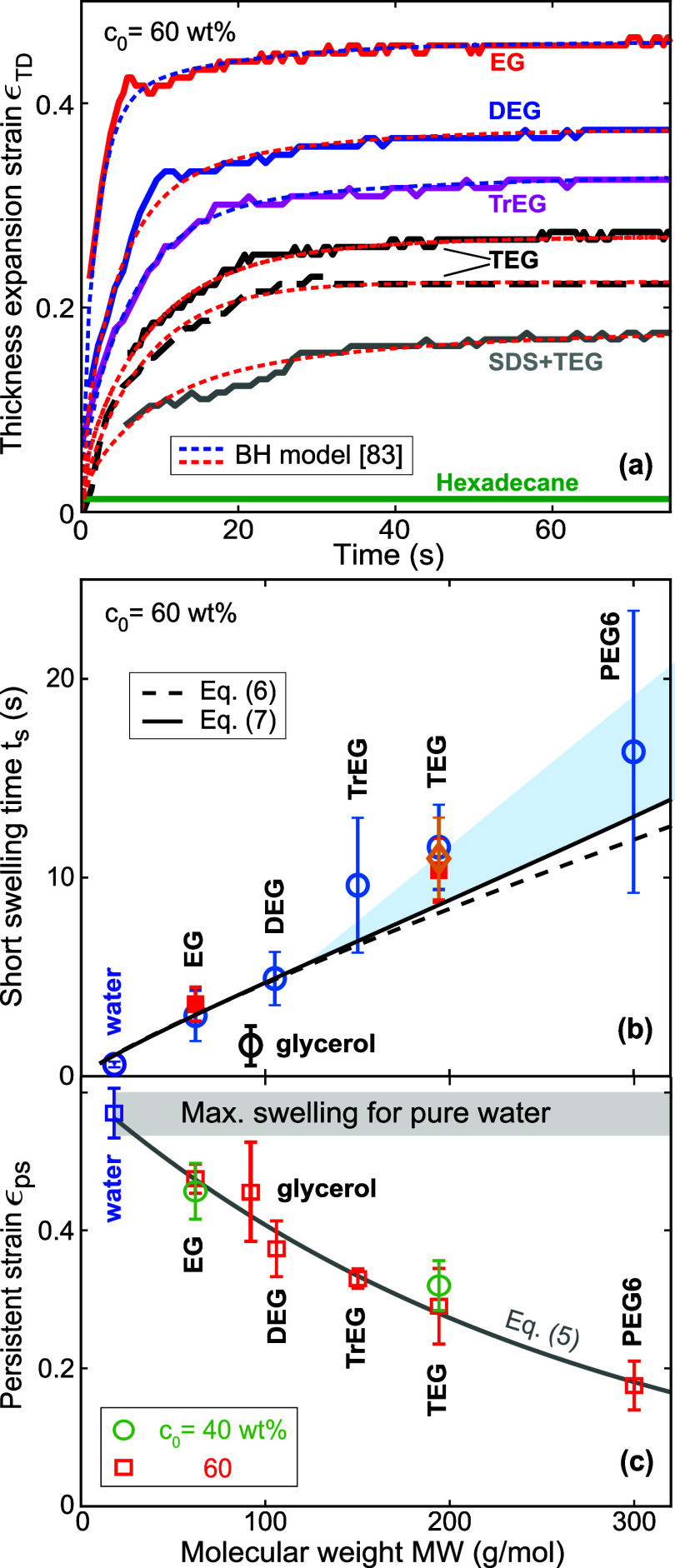
(a) Thickness expansion strain ϵ_TD_(*t*) for different cosolvents and paper types and a constant initial concentration *c*_0_ = 60 wt %. Solid lines correspond to paper A, the long-dashed line to paper B. The gray curve corresponds to a 60 wt % TEG solution containing 2.34 wt % SDS. The short-dashed lines are fits according to the Berens and Hopfenberg model. (b) Short swelling time *t*_s_ for water and aqueous cosolvent solutions as a function of molecular weight MW for a constant value of *c*_0_ = 60 wt %. Open circles correspond to paper A, solid red squares to paper B, the open orange diamond to paper C. The dashed and solid lines represent fitfunctions according to eqs 6 and 7 respectively. (c) Persistent strain ϵ_ps_ versus *MW* for water and aqueous cosolvent solutions with initial concentrations *c*_0_ = 60 wt % (red squares) and 40 wt % (green circles).

Figure 5a shows the thickness expansion strain ϵ_TD_ as a function of time for different cosolvents and paper types and a constant initial concentration *c*_0_ = 60 wt %. We also performed one experiment using pure hexadecane (green curve in Figure 5a), which is a nonpolar liquid. It does not induce any swelling of cellulose-based paper, although it readily imbibes into the interfiber pore space.

The corresponding short swelling times *t*_s_ are plotted in Figure 5b. They increase approximately linearly with cosolvent molecular weight. Within experimental reproducibility, the three paper types exhibit identical values of *t*_s_. The persistent strain ϵ_ps_ monotonically decreases with increasing MW as shown in Figure 5c. For comparison and as a reference value we have added the maximum strain for pure water. The solid line represents the fit function
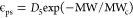
5with fit-parameters *D*_5_ = 0.61 and MW_c_ = 250 g/mol.

**Figure 6:**
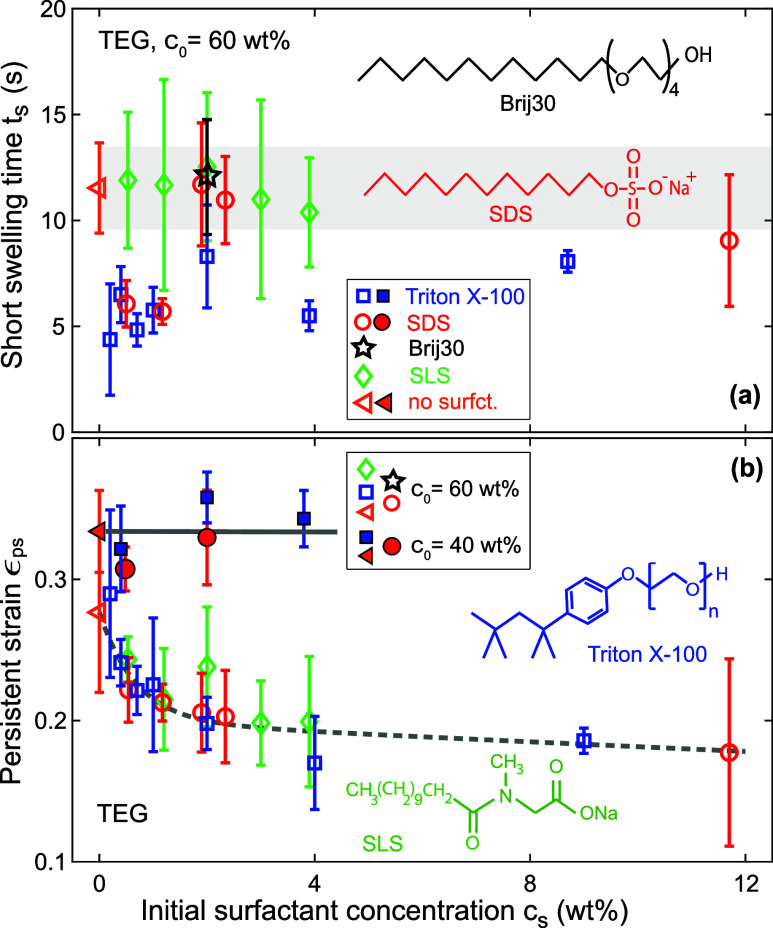
(a) Short swelling time *t*_s_ and (b) persistent strain ϵ_ps_ of paper A as a function of the initial surfactant concentration *c*_s_ for TEG solutions with SDS (red circles), TX-100 (blue squares), SLS (green diamonds), and Brij30 (black star). Solid and open symbols correspond to *c*_0_ = 40 and 60 wt %, respectively. Orange triangles denote surfactant-free solutions (*c*_s_ = 0). The gray lines are guides to the eye.

Figure 6a shows the dependence of the short swelling time *t*_s_ of paper A on the initial concentration *c*_s_ of the surfactants SDS, SLS, Triton X-100, and Brij30 in 60 wt % TEG solutions. Interestingly, *t*_s_ falls below the value of a surfactant-free 60 wt % TEG solution for SDS and Triton X-100 at small values of *c*_s_. According to Figure 6b, the persistent strain ϵ_ps_ monotonically decreases with *c*_s_ for *c*_0_ = 60 wt %, but remains constant for *c*_0_ = 40 wt %. Analogous data for aqueous surfactant solutions containing 40 wt % glycerol are available in Figure S9 of the Supporting Information.

### Validity – Imbibition Dynamics vs Pore-Fiber Transport

Measuring pore-fiber transport rates via the dynamics of swelling requires the imbibition time *t*_imb_ to be smaller than the swelling time *t*_s_. Nicasy et al. used a nuclear magnetic resonance (NMR) technique to investigate water transport in sheets of printing paper.^33^ They found an imbibition time scale of 0.07 s and a much larger swelling time scale of 0.7 s. For comparison, we found a very similar value of *t*_s_ = (0.6 ± 0.15) s for paper A. The requirement of imbibition outpacing swelling motivates our choice for uncoated, unsized and uncalendered paper types, as coatings, hydrophobization and calendering all tend to slow down imbibition.^34^

Aqueous cosolvent solutions have a markedly higher viscosity μ than pure water.^6^ Based on Darcy’s law, we expect the imbibition time to scale proportional to μ. Based on the model of Wang et al.,^8^ we expect *t*_s_ to scale proportional with μ as well, up to *c*_0_ ≈ 50 wt %. Beyond this concentration, an additional retardation effect is provided by the amount of water being insufficient to plasticize the cellulose fiber walls. Consequently, for the papers studied, the criterion of *t*_imb_ ≪ *t*_s_ is fulfilled for all cosolvents and all concentrations.

### Validity – Sheet Swelling vs Fiber Swelling

Inferring pore-fiber transport rates from measurements of the swelling dynamics of a papersheet also requires the latter to be primarily a consequence of the concerted swelling of the individual cellulose fibers. A conceivable alternative origin of swelling could be the loss of fiber–fiber bonds, the fibers becoming loose, leading to a loss of the cohesion of the paper structure. This scenario is reminiscent of hair being immersed in water. The key question in this context is thus whether fiber–fiber bonds are stable upon liquid deposition. The drastic reduction of mechanical stiffness and tear strength of paper upon water addition^35^ seems indeed to point toward a loss of stability. However, mechanical properties are typically assessed via stress–strain curves and therefore via application of external forces. In the case of ink deposition, no external forces are present that could lead to macroscopic relative displacements of individual fibers. Furthermore, Hirn and Schennach argue that the softening of the fibers might be an equally important contribution to the moisture-induced strength decrease of papersheets as the weakening of the fiber bonds.^36^

Two important mechanisms contributing to the fiber–fiber bond strength are interdiffusion and mechanical interlocking,^36−41^ which are not expected to disappear completely upon water addition. Moreover, Nanko and Ohsawa^42^ and Hobisch et al.^43^ showed that pulp fines predominantly aggregate at and near the fiber–fiber bond areas as do binders,^44^ adding another nonvanishing rheological component to the bond strength upon water addition.

Because the droplet imbibition time is shorter than the short swelling time in our experiments, the effective dry solids content does not fall below 60%. At this level, wet paper still maintains a non-negligible fraction (typically 3–10%) of its dry strength,^45−47^ again indicating that the interfiber bonds are weakened, but remain intact. Therefore, we believe that the scenario of a total loss of fiber cohesion is not relevant to our experiments.

A second reason why there could exist a sheet-swelling time scale that is unrelated to pore-fiber transport, is the relaxation of built-in drying stresses. It is conceivable that its primary origin is a weakening of the interfiber bonds (rather than the fibers becoming softer), which might not adhere to the same time scale as pore-fiber transport. We believe that the impact of drying-stress relaxation is similar in scale to the persistent strain ϵ_ps_ observed upon deposition and subsequent evaporation of pure water, which is generally on order of 2–3% in the thickness direction for papers A, B and C (see Supporting Information, Figure S7a–d). This is small compared to the maximum swelling amplitude, which is on order of 50%. The value of ϵ_ps_(100 wt % H_2_O) = 2–3% is consistent with literature values of the *in-plane* strain relaxation observed for restrained-dried paper upon cycling of the ambient relative humidity,^48−50^ assuming the expansion coefficient in the thickness direction is 20 times higher than in the cross-machine direction (CD).^7,51^ Moreover, we conducted additional experiments, where we first hydrated the paper sample twice with pure water and waited 2 h for unrestrained drying before depositing 1 μL of a TEG solution (*c*_0_ = 60 wt %). The purpose of the prehydration treatment was to remove any drying stresses present in the as-manufactured paper sample. The resulting values of *t*_s_ and ϵ_ps_ were identical to the ones in Figures 4 and 5 within error bars. Therefore, we believe that sheet-level drying stress relaxation does not invalidate the interpretation of our results toward pore-fiber transport, either.

### Effect of Cosolvent Concentration on Swelling Dynamics

Glycerol, poly(ethylene glycols) and cellulose have similar molecular structures containing polar alcohol and ether groups. This chemical compatibility allows cosolvents to penetrate the amorphous parts of cellulose fibers and thus induce swelling. Water plays a pivotal role in the pore-fiber transport dynamics of cosolvent solutions, as it acts as a plasticizer of the cellulose fiber walls and as it reduces the solution viscosity. Its presence in sufficient amounts therefore promotes the transport of cosolvents from pores to the fibers.^6,52^ Consequently, the swelling rate and the swelling amplitude induced by cosolvent solutions are expected to decrease with *c*_0_.

Both EG and TEG exhibit similar behavior in Figure 3, however the swelling time scales for TEG are longer. Moreover, max(ϵ_TD_) and the persistent strain ϵ_ps_ for TEG are lower than for EG. As we will show below and in the next subsection, these differences can be attributed to the difference in molecular weight (MW) of the two cosolvents.

For both cosolvents, increasing *c*_0_ results in more swelling and thus more cosolvent residing in the fibers up to a critical value *c*_0,crit_ ≈ 50 wt %.^6−8^ In equilibrium, this trend of ϵ_TD_ monotonically increasing with *c*_0_ continues up to *c*_0_ = 100 wt %.^7^ However, above *c*_0,crit_ the reduced water content in the solution restricts cosolvent transport and an equilibrium distribution is not attained before the water has evaporated. To illustrate that, we added a droplet of pure water (volume 1 μL) to the wet zone resulting from deposition of a 60 wt % TEG solution droplet (volume 1 μL) at *t* = 1000 s in Figure 3b. This rehydration step^6^ increased max(ϵ_TD_) and the persistent strain by about 75%.

The dashed lines in Figure 4a correspond to the fit functions
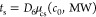
6and the solid ones to
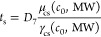
7Here, γ_cs_ and μ_cs_ are the (concentration dependent) surface tension and the viscosity of the cosolvent solutions^8^ and *D*_6,7_ are fit parameters. They reproduce the experimental data increasingly well, which indicates that viscosity is the dominant factor, but the effect of surface tension is non-negligible. The scaling of eq 7 with the liquid material parameters is the same as in Washburn’s or Darcy’s law, which indicates that capillary wicking is the dominant transport mechanism governing the short swelling time scale. A remaining difference to the experimental data – indicated by the shaded areas in Figure 4 – becomes apparent for *c*_0_ ≳ 50 wt %. This difference, which increases with *c*_0_, is ascribed to the incomplete or retarded plasticization of the cellulose fibers for low water contents.^8^

### Influence of Molecular Weight

In the previous subsection, we concluded that viscosity and surface tension are the key material parameters that determine the pore-fiber transport rates. The dashed and solid lines in Figure 5b correspond to eqs 6 and 7, respectively. They represent the experimental data well up to DEG. Because surface tension is a strong function of *c*_0_, but a weak function of MW for aqueous PEG solutions (see Figure S1a in the Supporting Information), the relative difference between the dashed and solid lines in Figure 5b is smaller than in Figure 4. The viscosity of aqueous PEG solutions is well represented by a power law μ_cs_ ∼ (MW)^β^. For *c*_0_ = 60 wt %, the exponent β ≈ 0.81 is close to 1 (see Figure S1b in the Supporting Information). The ratio of μ_cs_/γ_cs_ thus scales almost perfectly linearly with MW.

The shaded region in Figure 5b highlights the deviation of the data points from the Darcy-scaling represented by eq 7, which starts to be noticeable above MW ≈ 130. We expect that the dependence of *t*_s_ on MW eventually diverges as MW approaches the cutoff value, beyond which solute molecules are excluded from the intrafiber pores.^53,54^ Reported cutoff MW values are in the range of 250–5000.^55−57^

### Influence of Surfactants

Addition of surfactants to aqueous cosolvent solutions affects the solution viscosity only weakly,^58,59^ with the exception of Brij30, see Table 3, which contains values of the surface tension γ and viscosity μ corresponding to some of the data points in Figure 6. In contrast, the surface tension is reduced substantially by approximately 25–55% for surfactant concentrations close to or above the critical micelle concentration (cmc), see Table 3. According to eq 7, one would expect that the swelling time *t*_s_ should *increase* by a corresponding factor above the cmc.^60^ However, the experimental data in Figure 6a show a marked *decrease* of *t*_s_ below the value of a surfactant-free 60 wt % TEG solution for small values of *c*_s_ for SDS and TX-100. We ascribe this phenomenon to the reduction of the wetting delay by surfactant solutions, which is likely related to the expedited displacement of air that fills the pore space prior to ink imbibition.^61^ According to Fowkes^62^ and Cohen and Rosen,^63^ the wetting delay time of aqueous surfactant solutions on cotton to good approximation scales as *t*_wd_ ∼ *c*_s_^–β^ for concentrations below the cmc. Therefore, we expect the biggest reduction of *t*_s_ at or above the cmc.

**Table 3:** Surface Tension γ_cs_ and Viscosity μ_cs_ of Aqueous 60 wt % TEG Solutions as a Function of Surfactant Concentration *c*_s_ for a Temperature of (22.8 ± 0.3) °C

surfactant	*c*_s_ (wt %)	γ_cs_ (mN/m)	μ_cs_ (mPa s)
	0	51.3 ± 0.2	10.6
SDS	0.5	44.9 ± 0.2	10.7
SDS	1.9	39.9 ± 0.2	11.3
SDS	2.34	39.3 ± 0.2	11.2
SLS	1.2	35.9 ± 0.2	11.8
SLS	2	32.8 ± 0.2	11.6
TX-100	0.2	32.4 ± 0.3	8.7
TX-100	0.4	31.2 ± 0.3	9.5
TX-100	2	32.7 ± 0.2	12.0
Brij30	2	28.4 ± 0.2	22.3

In pure water, the cmc of SDS is 0.234 wt %, that of SLS is 0.4 wt %, and that of Triton X-100 is 0.026 wt %.^64−67^ For both anionic surfactants such as SDS and nonionic surfactants such as Tween and Triton X-100, the cmc increases with increasing cosolvent concentration.^68−75^ Based on the results of Ye et al.,^69^ we estimate the cmc of TX-100 in a 60 wt % TEG solution to be approximately 10 times higher than in pure water, i.e., around 0.26 wt %. According to Dey et al.,^75^ the cmc of SDS in a 60 wt % EG solution is approximately 4 times higher than in pure water, i.e., around 1.0 wt %.

Figure 6b shows that addition of surfactants does not affect ϵ_*∞*_ for *c*_0_ = 40 wt %. In contrast, surfactants decrease the persistent strain ϵ_ps_ compared to a surfactant-free, 60 wt % TEG solution. This indicates that a smaller fraction of the cosolvent reaches the interior of the cellulose fibers for *c*_0_ = 60 wt %.

### Origin of Two Swelling Time Scales

Solvent transport due to capillary imbibition into a porous medium or due to diffusion with a constant diffusion coefficient is characterized by Washburn or Fickian dynamics, which would give rise to a single swelling time scale. In our experiments in Figures 2, 3 and 5a, we frequently observe the presence of a shorter and a longer time scale. This is not uncommon and has also been observed e.g., in the swelling of starch granules by water,^76−78^ hydrogels,^79,80^ cellulose-based materials,^81−85^ and polymers.^86−94^

The occurrence of more than one time scale indicates that there is more than one mechanism at play. Berens and Hopfenberg (BH) devised an empirical model that accounts for solvent transport in polymers based on a combination of Fickian diffusion and polymer relaxation processes.^95^ The latter are ascribed to relatively large scale segmental motions of the polymer chains that are facilitated by the progressive swelling of the polymer matrix due to the increase in solvent content. Although for pore-fiber transport in cellulose fibers, Darcy flow might be dominant over diffusive transport, they both give rise to similar dynamics. The black dotted lines in Figure 3 and the dashed lines in Figure 5a are fits according to the BH model, which generally match the data very well.

In our experiments, we consider aqueous cosolvent solutions, i.e., mixtures of solvents with disparate molecular weights. The BH model takes only a single solvent into account. The time dependence of ϵ_TD_ for 80 and 100 wt % TEG solutions in Figure 5b is rather linear. This is reminiscent of case II sorption, where typically the effective transport coefficient strongly increases with solvent content, the solvent substantially swells the polymer and sharp penetration fronts are observed.^96^ Hermans and Vermaas^97^ observed the occurrence of a propagating sharp sorption front after immersion of a dry cellulose filament into water. For an aqueous 38 wt % glycerol solution, two separate fronts were observed. They interpreted the second as a glycerol front penetrating the region that has previously been plasticized by the passing of the first water front. It is unclear whether such sharp fronts also occur in chemically and mechanically processed (beaten) cellulose fibers as present in commercial printing paper. In the context of the BH model, the observed linear time dependence of ϵ_TD_ for 80 and 100 wt % TEG solutions can be reproduced by a substantially longer time scale *t*_l_ ≈ (1000–10000) s.

### Interpretation of WLI-Based Swelling Measurements

While the swelling times determined with the two methods agree well in Figure 4, the swelling amplitude observed with WLI in Figure 2 is about 50% smaller compared to that observed with microscopy-based thickness monitoring in Figure 3a. By design, WLI is a surface-metrology method. When applied to paper, usually a sparse data set results due to the considerable surface roughness. The full-width-at-half-maximum (FWHM) of the histogram corresponding to dry paper is 10.8 μm. Interestingly the FWHM of wet paper (16.5 μm) is larger than (1 + ϵ_TD_)(FWHM)_dry_. This might be an indication that the increase in transparency and reduction in scattering due to the presence of liquid^6^ allows subsurface locations to contribute to the WLI signal. This would result in an artificial reduction in swelling amplitude. Such an effect could explain the difference between the WLI and microscopy-based swelling amplitude measurements. The same observation holds for other optical methods based on light reflection such as confocal displacement metrology and laser triangulation, as reported in the Supporting Information (Section III, Figures S5 and S6).

## Conclusions

We have studied pore-fiber transport and the associated swelling dynamics of paper substrates after deposition of aqueous solutions of glycerol and poly(ethylene glycols). The latter are commonly added as cosolvents to water-based inks for inkjet printing. Typically we observe a short [*t*_*s*_ ≈ (1–20) s] and a long [*t*_*l*_ ≈ (20–10000) s] time scale. The short time scale depends approximately exponentially on the concentration and approximately linearly on the molecular weight of the cosolvents.

For cosolvent concentrations up to about 50 wt %, the short time scale is governed by capillary imbibition into the cellulose fibers, whereas at higher concentrations, it slows down due to the retarded or lacking plasticization of the fiber walls by water. The experimental data can be well-represented by the Berens and Hopfenberg model, according to which the long time scale represents the influence of polymer relaxation as a consequence of the increasing solvent content.

Glycerol has a similarly low vapor pressure as TEG at room temperature,^98−100^ but a significantly smaller short swelling time scale compared to TEG, which is likely related to the smaller molecular weight, higher surface tension and lower viscosity for concentrations below 50 wt %. As such it might lead to faster ink fixation compared to TEG.

Addition of surfactants can significantly reduce the short time scale. However, at high cosolvent concentrations, they tend to reduce the persistent swelling strain and thus the total quantity of cosolvent transported into the fiber interior.

We used optical noncontact methods that are compatible with the low mechanical stiffness and strength of wet paper. Methods based on light reflection from the top surface of paper were found to yield swelling amplitudes that are consistently smaller than optical monitoring of the cross-section of a papersheet. The reason is likely related to the finite penetration depth of light along the thickness direction of the paper sheet.

## Supporting Information Available

Material properties of the pure cosolvents and aqueous mixtures; experimental setups and results based on laser triangulation and confocal displacement metrology; additional results concerning the short swelling time: dependence on drop volume, DEG concentration and surfactant concentration for 40 wt % glycerol solutions (PDF)
