# Supplementary material for: Pore-Fiber Transport Dynamics of Aqueous Cosolvent Solutions in Paper
Source: Langmuir. 2024 Sep 3;40(37):19528–37. doi: 10.1021/acs.langmuir.4c01965 (PMC11411709; doi:10.1021/acs.langmuir.4c01965)
Supplement: Supplementary file 1 — la4c01965_si_001.pdf [file la4c01965_si_001.pdf]

Supporting information for manuscript: Pore-fiber transport dynamics of aqueous co-solvent solutions in paper

Sajjad Karimnejad,[1] Elian Gonnet,[1] Shuo Wang,[1] Hamid Mansouri,[2] Nicolae Tomozeiu,[2] and Anton A. Darhuber[1, a)]

[1)] *Department of Applied Physics, Eindhoven University of Technology*
[2)] *Canon Production Printing, Venlo, The Netherlands*

(Dated: 21 August 2024)

CONTENTS

I. Material properties of co-solvent solutions 1

II. Experimental methods 2
A. Laser triangulation metrology 2
B. Confocal displacement metrology 2

III. Experimental results 2
A. Laser triangulation metrology 2
B. Confocal displacement metrology 3
C. Microscopy-based thickness monitoring 3

I. MATERIAL PROPERTIES OF CO-SOLVENT SOLUTIONS

Table S1 lists the material properties of the pure co-solvents used in this study. Figure S1(a) shows surface tension γ_{cs} values measured with a Wilhelmy plate (red circles) of aqueous poly(ethylene glycol) solutions for different molecular weights MW and a constant initial co-solvent concentration of $c_0 = 60\,\mathrm{wt\%}$. Figure S1(b) reports data of the viscosity[1,3,4] μ_{cs} (red circles) of aqueous poly(ethylene glycol) solutions as a function of molecular weight MW for a constant initial co-solvent concentration of $c_0 = 60\,\mathrm{wt\%}$. The solid line in Fig. S1(a) corre-

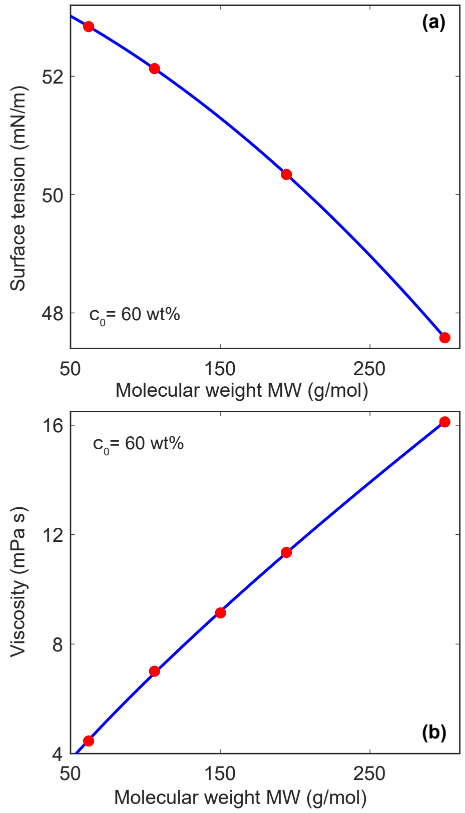

FIG. S1. (a) Surface tension and (b) viscosity of aqueous poly(ethylene glycol) solutions as a function of molecular weight. The co-solvent concentration was kept constant at $c_0 = 60\,\mathrm{wt\%}$. The filled red circles in (a) represent experimental data and those of (b) are from Refs. [1–4]. The solid lines are fit curves according to Eqs. (1,2).

[a)] Electronic mail: a.a.darhuber@tue.nl

sponds to a polynomial fit function

$$\gamma_{\rm cs}[{\rm mN/m}] = D_8(MW)^2 + D_9 MW + D_{10} \quad (1)$$

with fit parameters $D_8 = -3.02 \cdot 10^{-5}$, $D_9 = -0.011$ and $D_{10} = 53.65$. The values of MW have to substituted in units of g/mol. The solid line in Fig. S1(b) corresponds to a powerlaw fit function

$$\mu_{\rm cs}[{\rm mPa\,s}] = D_{11}(MW[{\rm g/mol}])^{\beta} \quad (2)$$

with fit parameters $D_{11} = 0.158$ and $\beta = 0.81$.

II. EXPERIMENTAL METHODS

Figure S2 shows the suction plate used for white-light interferometry, laser triangulation and confocal displacement metrology experiments. It consists of a polished Al plate with a $4 \times 4\,{\rm mm}^2$ hole array (hole diameter approximately $90\,\mu$m). A step of height $100\,\mu$m (close to the the thickness values $d_{\rm sub}$ of the papers A and B) was machined into the plate to serve as a reference surface for the WLI experiments. The throttle valve was adjusted such that the underpressure in the vacuum chamber was kept at the minimum necessary to keep the paper flat.

A. Laser triangulation metrology

Laser triangulation is a technique for measuring surface displacements. Figure S3 illustrates its operating principle. A laser beam is projected onto the target surface to form a laser spot, which is imaged onto a line detector. From the image location, the vertical position of the target surface can be reconstructed.

B. Confocal displacement metrology

Confocal displacement metrology is another measurement method for vertical positions and displacements of a

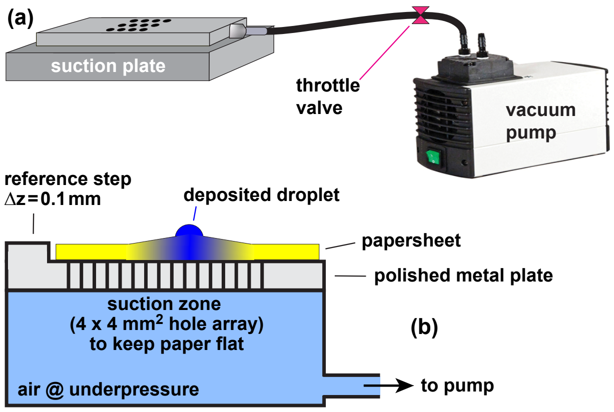

FIG. S2. (a) Schematic representation of the suction plate setup. (b) Vertical cross-section through suction plate.

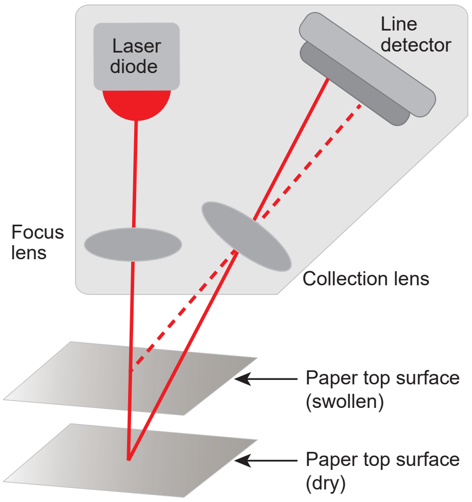

FIG. S3. Operating principle of laser triangulation metrology.

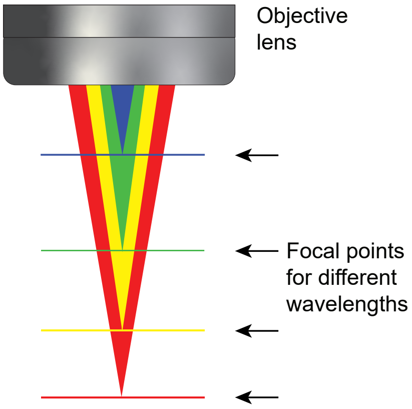

FIG. S4. Operating principle of confocal displacement metrology.

reflective surface. Figure S4 illustrates its operating principle. Due to chromatic aberration, the different spectral components of a white-light beam are focused at different focal planes below the objective. In fact, this information is encoded into spectral characteristics of the light reflected from the sample. The spectrum of the light on the basis of the wavelength dependence of longitudinal chromatic aberration will provide enough information over the depth scanning. It is worth mentioning that the small measurement spot size enables detecting small objects and most importantly can be useful for even diffuse and reflecting objects.

III. EXPERIMENTAL RESULTS

A. Laser triangulation metrology

Figure S5 shows example data of the thickness swelling amplitude for aqueous solutions of TEG on paper A obtained with laser triangulation using a Micro Epsilon

Co-solvent	product#	MW	μ_{cs} [mPa s]	γ_{cs} [mN/m]	ρ_{cs} [kg/m^3]
Glycerol	449770	92.1	1206	63.5	1258
Ethylene glycol (EG)	324558	62.1	19.8	48.0	1110
Diethylene glycol (DEG)	32160	106.1	35.7	44.8	1118
Triethylene glycol (TrEG)	90390	150.2	49.0	45.5	1120
Tetrathylene glycol (TEG)	110175	194.2	58.3	44.0	1121
Polyethylene glycol 300 (PEG6)	49770	300±15	91.0	-	1125

TABLE S1. Material properties of pure co-solvents. The values of viscosity μ_{cs} and surface tension γ_{cs} are given for temperatures of 20°C and 25°C, respectively.

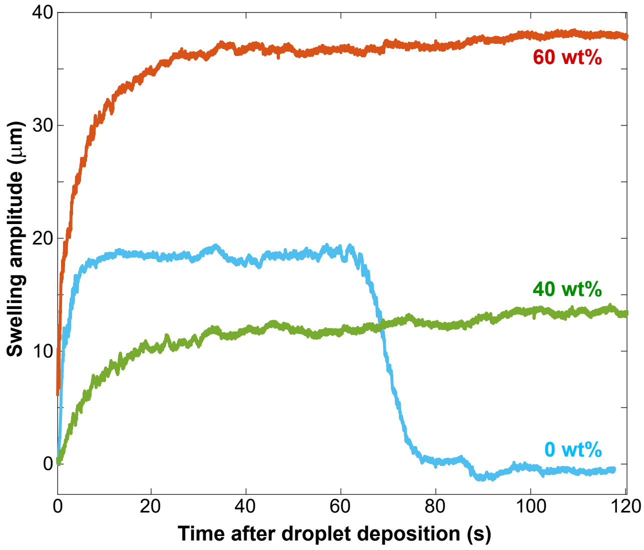

FIG. S5. Thickness swelling amplitude of paper A as a function of time after deposition of aqueous solutions of TEG measured by laser triangulation.

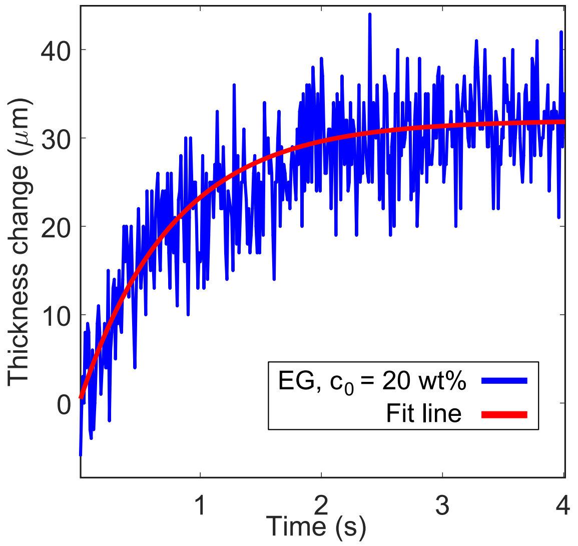

FIG. S6. Thickness expansion as a function of time for an aqueous solution of EG ($c_0 = 20\,$wt%) in paper A monitored by confocal displacement metrology.

optoNCDT ILD2300-10BL system operating at a wavelength of 405 nm. Frequent issues are baseline drift and erratic signals. The swelling amplitude for pure water in Fig. S5 is about a factor of 3 lower than observed with microscopy-based thickness monitoring.

B. Confocal displacement metrology

During the experiment, a droplet of co-solvent solution (volume $2\,\mu$l) is deposited onto the paper sample using a Hamilton digital syringe close to the optical axis of the sensor. In this fashion, fast spreading and imbibition towards the measurement spot is ensured, while the droplet itself does not obstruct the measurement. We used a Micro Epsilon IFS2405-10 system.

As the paper sample expands in thickness, the surface displacement is recorded and the thickness change is calculated. Fig. S6 shows an example of the swelling dynamics for an aqueous EG solution with initial concentration $c_0 = 20\,$wt%. The red solid line is a fit according to

$$\Delta z = D_{12}[1 - \exp(D_{13} t)], \qquad (3)$$

with fit parameters $D_{12} = 32\,\mu$m and $D_{13} = -1.3\,$s^{-1}. The noise amplitude is on order of $\pm 5\,\mu$m. Unfortunately, the method did not work for higher values of c_0. Our hypothesis is that since glycols are very nearly refractive-index-matched to cellulose, the surface reflectivity of the wet paper dropped and its transmission increased too much for the sensor to accurately detect the location of the top surface.

C. Microscopy-based thickness monitoring

Figure S7(a-d) shows the thickness expansion strain ϵ_{TD} as a function of time for different droplet volumes V_{drop} of pure water. Figure S7(e,f) illustrates the maximum strain and drying time as a function of V_{drop}. We conclude that the chosen value of $V_{drop} = 1\,$µl is in a range where the expansion strain and thus the pore-fiber transport do not depend on V_{drop}. As long as

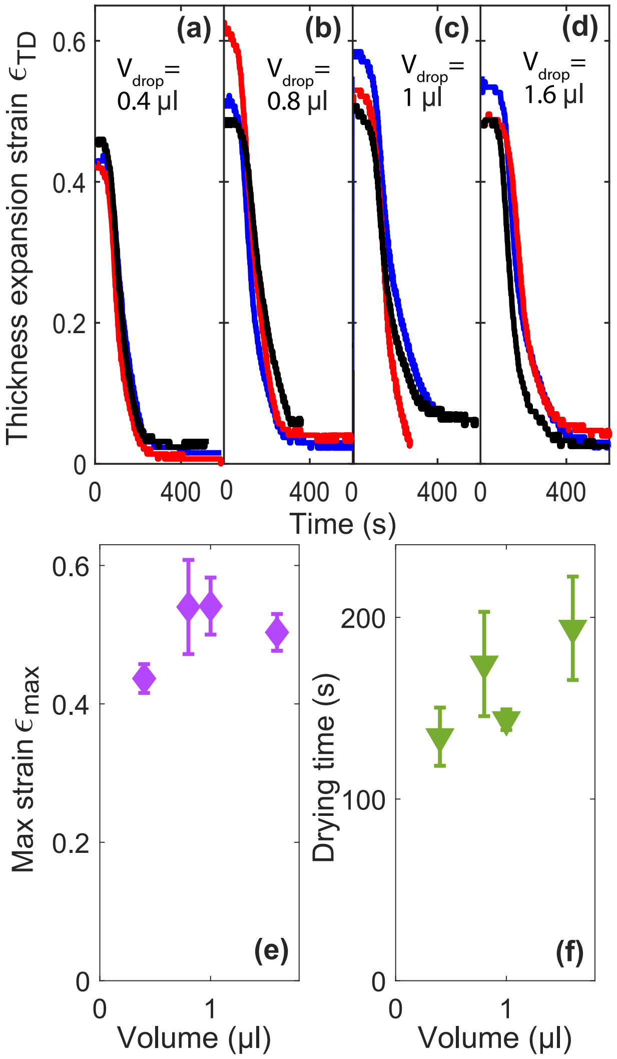

FIG. S7. Effect of droplet volume on thickness expansion strain and drying time for pure water. (a-d) Thickness expansion strain ϵ_{TD} as a function of time for different droplet volumes $V_{drop} = 0.4, 0.8, 1$ and $1.6\,\mu l$, respectively. (e) Maximum strain and (f) drying time as a function of V_{drop}.

any timescale of interest is shorter than the drying time ($t_{dry} \approx 150\,s$ for $V_{drop} = 1\,\mu l$), the solvent evaporation and the corresponding change in solution concentration and viscosity do not affect the pore-fiber transport significantly.

Figure S8 shows the short swelling time t_s and long swelling time t_l as a function of the initial co-solvent concentration c_0 for aqueous DEG solutions. The behavior is qualitatively analogous to that of EG and TEG (see Fig. 4 in the manuscript).

Figure S9 shows the short swelling time t_s and persistent strain ϵ_{ps} of paper A as a function of the initial surfactant concentration c_s for aqueous solutions of glycerol ($c_0 = 40\,wt\%$) containing either SDS or Triton X-100.

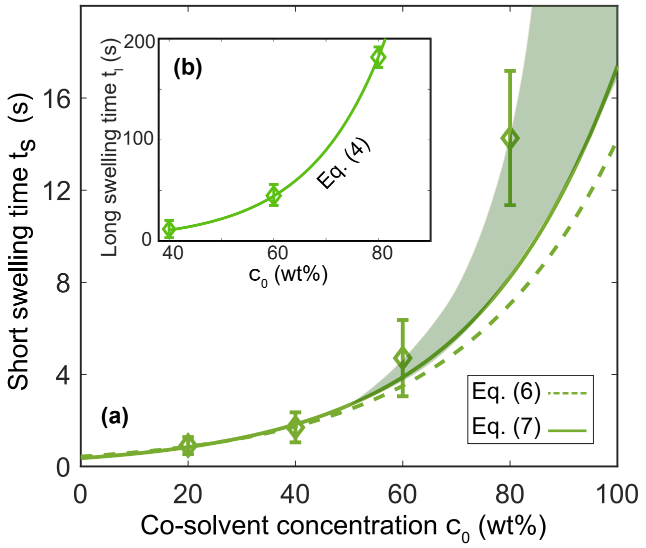

FIG. S8. (a) Short swelling time t_s of paper A as a function of initial concentration c_0 of DEG (green diamonds). The dashed and solid lines represent fit functions according to Eqs. (6) and (7) of the main manuscript, respectively. (b) Long swelling time t_l of paper A as a function of c_0 for DEG. The solid line represents the fit function according to Eq. (4) of the main manuscript.

[1] *MonoEthylene Glycol product guide.* MEGlobal, 2008.
[2] C. I. A. V. Santos, M. C. F. Barros, A. C. F. Ribeiro, M. M. Bou-Ali, A. Mialdun, and V. Shevtsova. Transport properties of n-ethylene glycol aqueous solutions with focus on triethylene glycol–water. *J. Chem. Phys.*, 156:214501, 2022.
[3] *Tetraethylene Glycol.* The Dow Chemical Company, 2003.
[4] *Diethylene Glycol product guide.* MEGlobal, 2014.

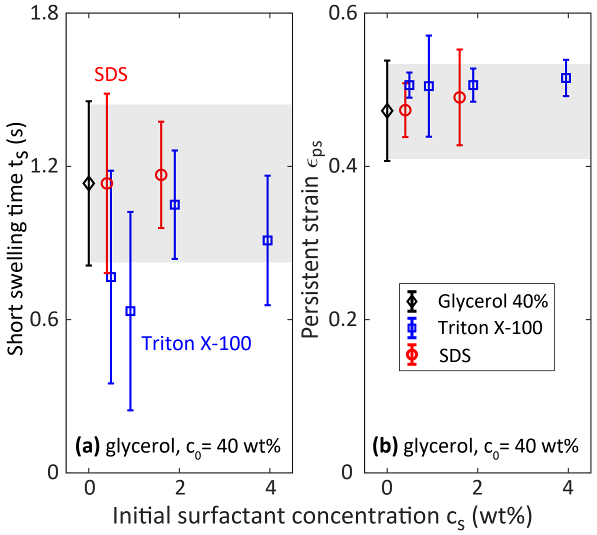

FIG. S9. (a) Short swelling time t_s and (b) persistent strain ϵ_{ps} of paper A as a function of the initial surfactant concentration c_s for aqueous 40 wt% solutions of glycerol with added SDS (red circles) and Triton X-100 (blue squares).
